# New protocol for early robot-assisted gait training after spinal surgery

**DOI:** 10.3389/fmed.2024.1450883

**Published:** 2024-10-23

**Authors:** Sanghyun Jee, Chan Woong Jang, Sang Hoon Shin, Yeji Kim, Jung Hyun Park

**Affiliations:** ^1^Department of Rehabilitation Medicine, Gangnam Severance Hospital, Rehabilitation Institute of Neuromuscular Disease, Yonsei University College of Medicine, Seoul, Republic of Korea; ^2^Department of Physical Medicine and Rehabilitation, Hallym University Sacred Heart Hospital, Hallym University College of Medicine, Anyang, Republic of Korea; ^3^Command Center, Doheon Institute for Digital Innovation in Medicine, Hallym University Medical Center, Anyang, Republic of Korea; ^4^Department of Physical Medicine and Rehabilitation, National Health Insurance Service Ilsan Hospital, Goyang, Republic of Korea; ^5^Department of Medical Device Engineering and Management, The Graduate School, Yonsei University College of Medicine, Seoul, Republic of Korea; ^6^Department of Integrative Medicine, The Graduate School, Yonsei University College of Medicine, Seoul, Republic of Korea

**Keywords:** enhanced postoperative recovery, exercise therapy, patient satisfaction, rehabilitation, robotics, spine

## Abstract

**Introduction:**

Early rehabilitation post-spinal surgery is vital for patients' recovery. Robot-assisted gait training (RAGT) shows promise but requires further study to establish a specific protocol and gauge its effects on both patients and physical therapists. This study aimed to determine the impact of a newly developed protocol for early RAGT on patients' functional enhancement and satisfaction levels after spinal surgery, as well as on the physical therapists who implemented the therapy.

**Methods:**

First, we developed the protocol in collaboration with three physiatrists and two physical therapists with extensive experience in musculoskeletal rehabilitation. The protocol was updated three times, each after three rounds of face-to-face meetings. Afterward, we conducted a cross-sectional study involving five physical therapists and 32 post-spinal surgery patients at a tertiary hospital rehabilitation center. The intervention consisted of five sessions of RAGT. Main outcome measures included the Functional Ambulation Category (FAC), the ambulation item of the Modified Barthel Index (MBI ambulation), and satisfaction surveys for both patients and physical therapists.

**Results:**

RAGT typically started 17.91 ± 9.76 days postoperatively and was successfully applied with no remarkable adverse effects. The FAC scores increased from 2.65 ± 1.21 to 3.78 ± 0.71 *(p* = 0.006), and MBI ambulation increased from 7.69 ± 2.71 to 10.66 ± 2.90 (*p* < 0.001) between transfer and discharge. Satisfaction with the robot, RAGT, and treatment, assessed using a 5-point Likert scale, were 3.30 ± 0.79, 3.72 ± 0.85, and 3.08 ± 0.84, respectively. Satisfaction was notably the highest for alleviating fear of falling, whereas managing pain and discomfort during position changes scored the lowest. Physical therapists rated RAGT satisfaction, impact on the working environment, and treatment stability at 3.0 ± 0.65, 2.80 ± 0.67, and 3.50 ± 0.61, respectively.

**Conclusion:**

Early spinal surgery rehabilitation with RAGT improved patients' functionality and gait satisfaction. While physical therapists considered RAGT safe, its impact on their work environment was limited. Integrating RAGT into post-spinal surgery rehabilitation demands ongoing protocol refinement, custom robot development, and efficacy evaluations.

## 1 Introduction

The numbers of patients undergoing spinal surgery and of elderly persons eligible for surgical interventions are increasing (1, 2). Regardless of pathology and technique, a variable proportion of patients experience persistent postoperative symptoms and functional disability (3, 4). In this context, postoperative intensive rehabilitation plays a crucial role in optimizing spine care (5). It includes conventional physical therapy and facilitates a quicker recovery, by focusing on improving daily activities and achieving the goal of returning to work, sports, and leisure activities (6, 7).

The concept of enhanced recovery after surgery (ERAS) has recently gained traction in spinal surgery (8, 9). It represents an evidence-based, multidisciplinary approach to peri-operative management after major surgery (10) and has reduced complication rates after spinal procedures (11). Indeed, a recent consensus statement on ERAS for patients undergoing lumbar spinal fusion surgery strongly advocates early mobilization and postoperative in-hospital physical therapy (12). Despite these recommendations, standardized ERAS protocols for mobilization and physical therapy after spinal surgery have not been standardized. This suggests that the subjective opinions of healthcare providers and physical therapists can lead to variations in rehabilitation methods between institutions.

Effort has been directed toward providing early robot-assisted gait training (RAGT) to elderly patients after spinal surgery. A study of two elderly patients who participated in RAGT after posterior lumbar spinal fusion and minimally invasive transforaminal lumbar interbody fusion surgery, respectively, had good outcomes, with notable functional improvements and no significant adverse effects (13). Although this was only a case report, it highlighted the feasibility of early RAGT after spinal surgery. Furthermore, given that three other studies have also found RAGT effective in treating various conditions (14–16), RAGT may be a versatile modality that is both safe and effective for patients after spinal surgery. However, a limitation of these studies is that the RAGT protocols used were all different, which presents significant challenges when applying them to patients after spinal surgery.

Developing a treatment protocol is essential to promote the broader and more proactive adoption of this therapy and to provide evidence of its effectiveness. Additionally, conducting satisfaction surveys with participants regarding the new protocol is an excellent way to evaluate its applicability. Understanding the experience of physical therapists who delivering RAGT is also critical. Therefore, the primary objective of this study was to introduce a new RAGT protocol for post-spinal surgery and evaluate its effectiveness by assessing patients' functional improvements. The secondary objective was to gauge the satisfaction levels of both patients and physical therapists through surveys and assess the protocol's applicability.

## 2 Materials and methods

### 2.1 Study design

This cross-sectional study determined the effects of a newly developed protocol for RAGT on gait functionality in patients after spinal surgery. In addition, we explored satisfaction levels and the experiences of the patients and physical therapists using questionnaires. The Institutional Review Board of Gangnam Severance Hospital approved the study (IRB No. 3-2024-0050), and waived the need for written informed consent due to the retrospective design of the study. This study conforms to all STROBE guidelines and reports the required information accordingly (see Supplementary material).

### 2.2 Participants

This study involved 32 patients and five physical therapists. The inclusion criteria for patients were: (1) referral to the Department of Rehabilitation Medicine at a tertiary hospital for intensive postoperative rehabilitation, (2) having undergone spinal surgery between June and December 2023, (3) ability to sit independently and stand under supervision, (4) participation in RAGT, and (5) completion of the satisfaction survey. Ultimately, 32 patients who actively engaged in RAGT with the goal of restoring preoperative function were included in the study. Among the therapists who administered RAGT to these patients, six were identified during the specified period, and five who treated ≥5 patients completed the survey.

### 2.3 Robot-assisted gait training

**Figure 1:**
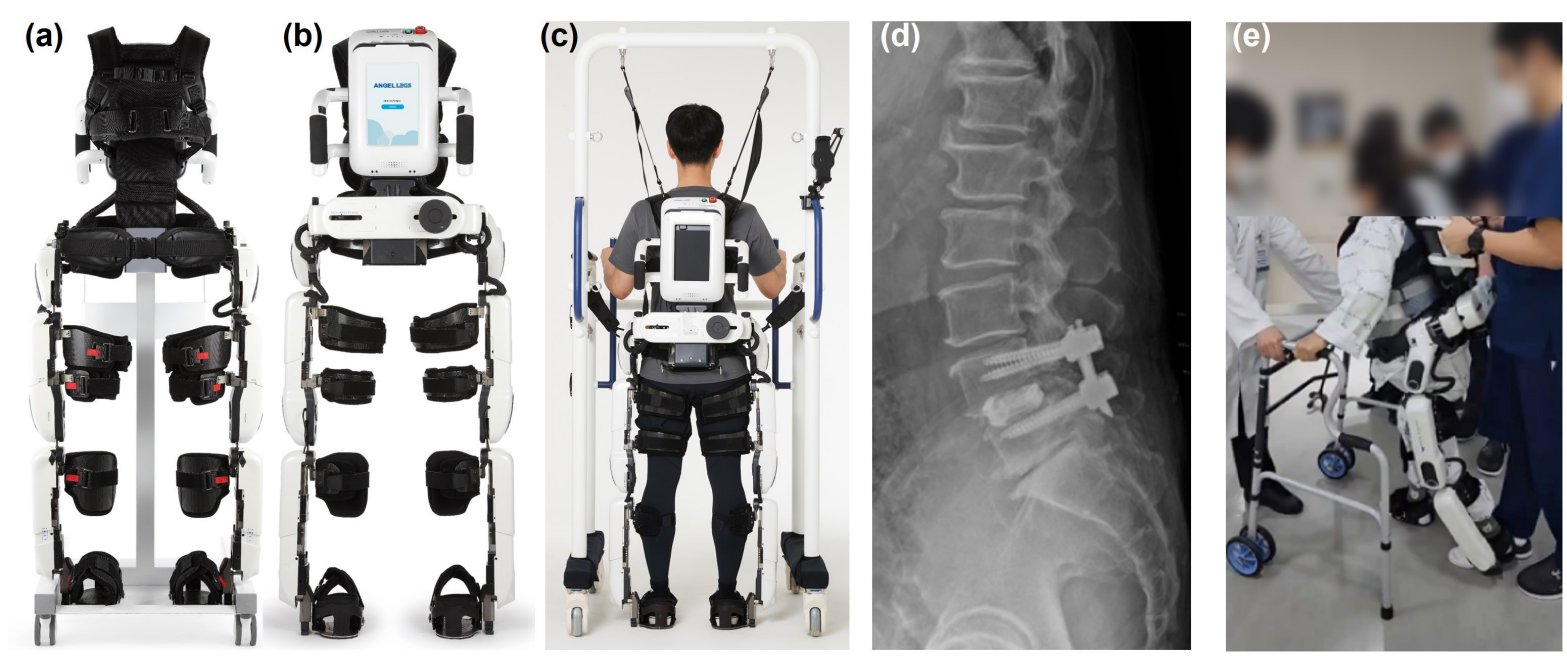
Exoskeletal wearable robot and patient who underwent RAGT. **(a)** Anterior and **(b)** posterior views of the robot. **(c)** Posterior view of a patient wearing the robot and using a harness. **(d)** Lateral lumbar radiograph and **(e)** image shows a patient undergoing RAGT while wearing a spinal orthosis.

The wearable exoskeletal robot ANGEL LEGS M20 (Angel Robotics, Seoul, Republic of Korea) was designed as an orthopedic exercise device for gait training (Figure 1). It consists of hip, knee, and ankle segments, and offers torque assistance at the hip and knee joints, which is automatically detected by force sensors beneath the ankle-foot-orthosis. Its actuators generate flexion torque during the swing phase and extension torque during the stance phase, thus promoting proper gait and providing lower limb support. None of the participants received other forms of robot-assisted rehabilitation.

At our hospital, patients are transferred to the Department of Rehabilitation Medicine as soon as possible after surgery to begin postoperative rehabilitation. Once transferred, they receive conventional physical therapy, and when they can sit independently and stand under supervision, we confirm their willingness to undergo RAGT. If the patient consents to start RAGT, they are comprehensively assessed beforehand using manual muscle tests, range of motion evaluations, and spasticity assessments in all extremities. Each patient is scheduled to receive at least five sessions of RAGT, each lasting 30 min, with one session per day. The start and progression of RAGT are tailored to each patient's functional level, with assistance reduced as functionality improves, allowing for more advanced movements. During these sessions, a physical therapist administers gait training using an exoskeleton, following the new protocol that accounts for the time required to attach and detach the robot.

Patients also continue conventional physical therapy during RAGT, with five 30-min sessions per week. This treatment is performed before RAGT, and the time interval between conventional therapy and RAGT is adjusted according to the patient's schedule.

In the event of an unexpected gear response disrupting the gait rhythm or causing fatigue or discomfort, the participants could use an emergency switch to halt gait control and power assistance. Comprehensive training on emergency device removal was provided to the participants and therapists to mitigate the risk of falls and potential musculoskeletal injuries. A fall-proof harness was also supplied to enhance safety during gait training.

### 2.4 RAGT protocol

Table 1 shows the new treatment protocol that was customized for gait training in patients after spinal surgery. It comprises four gait assistive algorithms: stand-up (from a chair), standing, walking, and up-and-down stair modes; all were based on passivity-guaranteed control to ensure safety. We developed the protocol in a collaboration with three physiatrists and two physical therapists with extensive experience in musculoskeletal rehabilitation. The protocol was updated three times, each after three rounds of face-to-face meetings. Supplementary Tables 1–3 describe previous versions and the changes applied. The version applied herein is the most recent, and the protocol remains under continuous development.

**Table 1:** Protocol for early robot-assisted gait training (RAGT) after spinal surgery.

**Mode**	**Task type**	**Stage 1**	**Stage 2**	**Stage 3**
A	Basic	Stand for 30 s (stand mode)		
	Additional	None	20 squats (squat mode)	> 20 squats with slow sit down (squat mode)
B	Basic	Stand for 1 min (stand mode)		
	Additional	Weight shift alternately lifting hands (stand mode)	Weight shift alternately lifting feet (stair mode)	Weight shift lifting one foot (stair mode)
C	Basic	Even level gait (gait mode)		
	Additional	(Harness or walker) steps: 200–400 cadence: < 50 [crutch] steps: 600–800 cadence: >70	(Harness or walker) steps: 400–600 cadence: < 65 [crutch] steps: >800 cadence: >75	Uneven level gait or up-and-down stair training (gait mode and up-and-down stair mode)

It took ~5 min to don and doff the robot. Each session, apart from the time required to attach and detach the robot, involves A mode + (B or C mode), totaling 25 min. However, the selected modes and stages can vary depending on the condition of each patient. In modes B and C, three assistive devices (harness with an anterior walker, anterior walker alone, crutches) can be used based on functional status of each patient.

### 2.5 Measures

We evaluated functional improvement in the patients and satisfaction in both patients and the therapists. Functional improvement was assessed using the Functional Ambulation Category (FAC) and the ambulation item of the modified Barthel index (MBI ambulation) at the time of transfer to and discharge from the rehabilitation center.

Furthermore, we assessed the satisfaction of patients who received RAGT and self-reported adverse events using questionnaires distributed and collected after the final treatment session by the physical therapists. Patients who received RAGT at least once were encouraged to complete a written survey to gather information about how satisfied physical therapists were with RAGT, including improvements in the working environment. Physical therapists could complete the questionnaire if they had administered RAGT to at least five patients. The questionnaires were developed based on the findings of a targeted literature review and discussions with authors (17, 18) (Table 2).

**Table 2:** Survey of robot-assisted gait training (RAGT) for patients (A) and physical therapists (B).

**A. Questions for patients**	
Robot	1. Did you find the robot easy attach?
	2. Was the robot comfortable?
	3. Do you consider the noise generated by the robot appropriate?
	4. Was the weight of the robot suitable in your perception?
RAGT	5. Did you easily adapt to RAGT?
	6. Do you believe that your quality of life improved after RAGT?
	7. Would you continue RAGT in the future?
	8. Would you recommend RAGT to others?
Effectiveness	9. Does RAGT has helped to improve your muscle strength and endurance?
	10. Does RAGT has improved your balance?
	11. Does RAGT has helped to alleviate pain?
	12. Does RAGT has helped to reduce discomfort when changing positions?
	13. Does RAGT has helped to diminish fear of falling?
	14. Does RAGT has improved your walking ability?
**B. Questions for physical therapists**	
	1. How satisfied do you feel with RAGT?
	2. Have you noticed an improvement in the working environment?
	3. Do you consider RAGT safe?
	4. Did you encounter accidents, treatment interruptions, or side effects during RAGT?

Responses to all questions were scored on a scale, where 1, 2, 3, 4, and 5 represent strongly disagree, disagree, neutral, agree, and strongly agree, respectively.

### 2.6 Statistical analyses

Descriptive statistics for all variables are shown as means ± standard deviation (SD), frequencies, and proportions. The significance of changes in functional levels before and after was assessed using Wilcoxon signed-rank tests. An exploratory analysis was conducted to identify potential differences in treatment satisfaction based on surgical levels and types. Differences among the three surgical level groups were assessed using Kruskal-Wallis H tests with Tukey *post-hoc* analysis, while differences between the two surgical type groups were evaluated using Mann-Whitney *U*-tests. All data were statistically analyzed using R version 4.1.2 (R Foundation for Statistical Computing, Vienna, Austria). Values with *p* < 0.05 were considered statistically significant.

## 3 Results

Tables 3, 4 outlines the basic characteristics and functional levels of 32 patients who participated in the survey. All patients started RAGT on an average of 17.91 ± 9.76 postoperative days (PODs). The main reason for spinal surgery was degenerative spinal diseases (*n* = 17; 53.13%), with lumbar level surgeries being the most common (*n* = 18; 56.25%). The average number of RAGT sessions received by the participants was 4.78 ± 0.83. The FAC and the MBI ambulation improved in patients from 2.65 ± 1.21 to 3.78 ± 0.71 (*p* = 0.006) and from 7.69 ± 2.71 to 10.66 ± 2.90 (*p* < 0.001), respectively, between transfer and discharge, as determined by Wilcoxon signed-rank tests.

**Table 3:** Basic characteristics of patients (*n* = 32).

**Characteristics**	**Patients**
Mean (±SD) age, years	66.75 ± 13.11
**Sex**, ***n*** **(%)**	
Men	11 (34.38%)
Women	21 (65.63%)
**Reason for surgery**, ***n*** **(%)**	
Degenerative^a^	17 (53.13%)
Trauma	6 (18.75%)
Tumor	3 (9.38%)
Infection	2 (6.25%)
Deformity	4 (12.50%)
**Surgical location**, ***n*** **(%)**	
Cervical	9 (28.13%)
Thoracic	5 (15.63%)
Lumbar	18 (56.25%)
**Surgical type**, ***n*** **(%)**	
Fusion	10 (31.25%)
Non-fusion	22 (68.75%)
**Mean (**±**SD) interval, days**	
Surgery to transfer^b^	11.81 ± 8.50
Transfer^b^ to start of RAGT	6.10 ± 5.23
Surgery to start of RAGT	17.91 ± 9.76
Mean (±SD) RAGT sessions, times	4.78 ± 0.83

SD, standard deviation.

^a^Includes spinal stenosis, degenerative disk diseases, degenerative spondylolisthesis.

^b^Refers to point when patients were transferred to Department of Rehabilitation Medicine for intensive postoperative rehabilitation.

**Table 4:** Functional levels of patients (*n* = 32).

**Functional levels**	**Transfer**	**Discharge**	***P*-value**
FAC, mean (± SD)	2.65 ± 1.21	3.78 ± 0.71	0.006
Score 0, *n* (%)	4 (12.50)	0 (0)	
Score 1, *n* (%)	1 (3.13)	0 (0)	
Score 2, *n* (%)	4 (12.50)	0 (0)	
Score 3, *n* (%)	17 (53.13)	12 (37.50)	
Score 4, *n* (%)	6 (18.75)	15 (46.88)	
Score 5, *n* (%)	0 (0)	5 (15.63)	
Ambulation item of MBI, mean (± SD)	7.69 ± 2.71	10.66 ± 2.90	< 0.001
Score 0, *n* (%)	0 (0)	0 (0)	
Score 3, *n* (%)	6 (18.75)	0 (0)	
Score 8, *n* (%)	21 (65.63)	16 (50.00)	
Score 12, *n* (%)	5 (15.63)	9 (28.13)	
Score 15, *n* (%)	0 (0)	7 (21.88)	

FAC, functional ambulation category; MBI, modified Barthel index; SD, standard deviation. *P*-value by Wilcoxon signed ranked tests.

**Figure 2:**
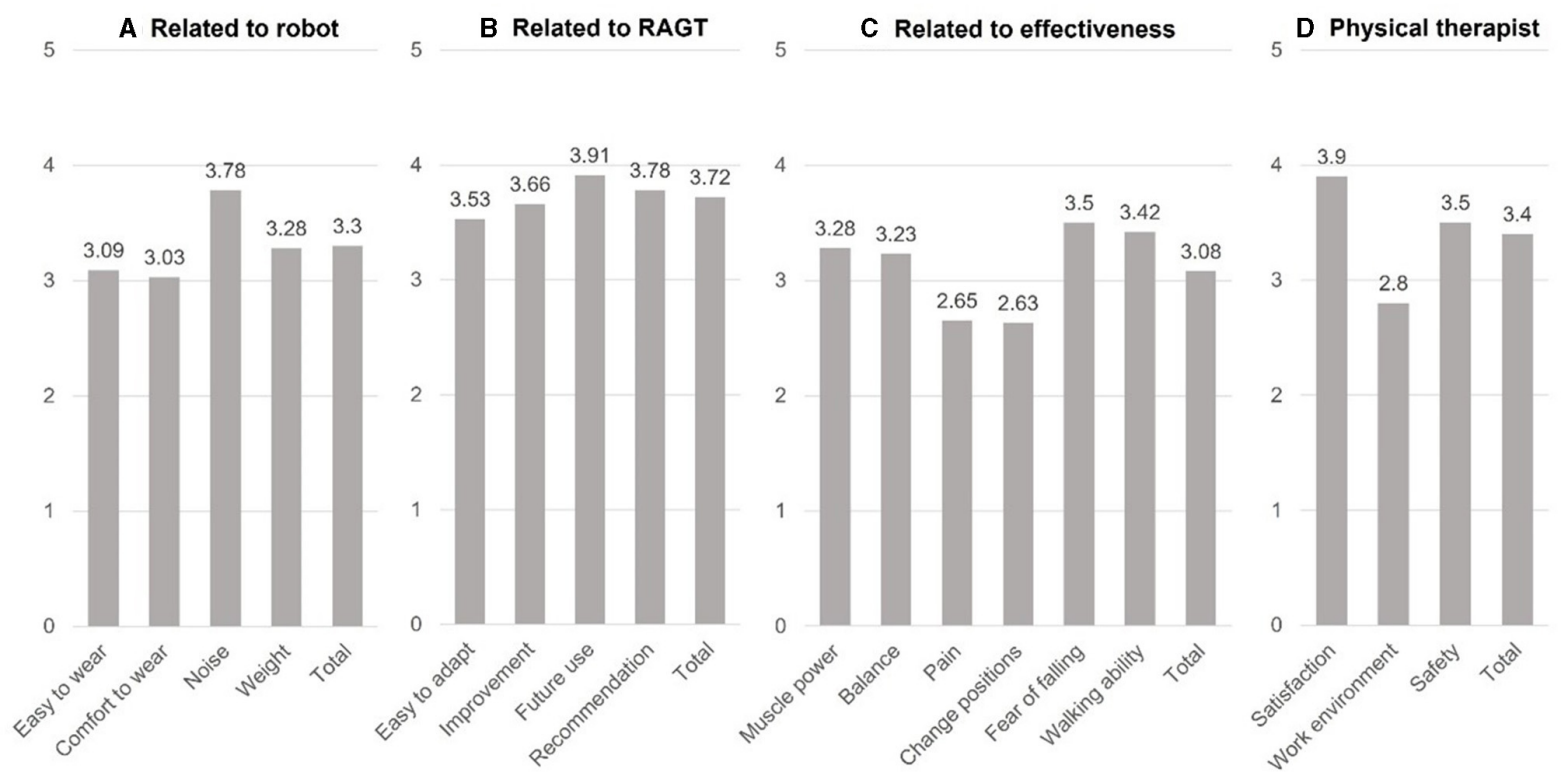
Average satisfaction survey results of patients **(A–C)** and physical therapists **(D)** 5-point scale. RAGT, robot-assisted gait training.

In terms of patient satisfaction measured on the 5-point Likert scale, the average score for the robot was 3.30 ± 0.79 (Figures 2A–C). Satisfaction with RAGT and its future application scored 3.72 ± 0.85 and 3.91 ± 1.06, respectively. The average score for satisfaction with treatment was 3.08 ± 0.84, with lower scores regarding pain relief and discomfort during postural changes. After completing each RAGT session according to the protocol, all patients reported mild fatigue, which naturally subsided within a few hours without raising any medical concerns, allowing us to continue the treatment as planned. Additionally, five patients reported muscle pain in the upper limbs, particularly in the shoulder and neck areas. This pain was mainly attributed to patients exerting excessive force with their arms to maintain balance, driven by fear of falling and discomfort during the treatment. The muscle pain was mild and manageable with pain relievers, enabling the continuation of the treatment. No serious side effects, such as worsened pain at surgical sites, deterioration of neurological symptoms, falls, or loss of consciousness, were reported. Furthermore, there were no incidents that necessitated the use of the emergency switch to halt the gait training.

Satisfaction rates related to “Robot,” “RAGT,” and “effectiveness” did not significantly differ among three subgroups groups on cervical, thoracic, and lumbar surgeries (*p* = 0.83, *p* = 0.11, and *p* = 0.48 by Kruskal-Wallis H tests, respectively). *Post-hoc* analysis using Tukey also did not reveal differences among these subgroups. Satisfaction rates also did not significantly differ between fusion and non-fusion subgroups of patients who underwent lumbar surgery level (“Related to: “Robot,” “RAGT,” and “effectiveness”; *p* = 1.00, *p* = 0.32, and *p* = 0.24 by Mann-Whitney *U*-tests, respectively).

According to assessments by physical therapists (Figure 2D), the rates of subjective satisfaction with RAGT, impact on improving the working environment and the stability of the treatment were 3.90 ± 0.65, 2.80 ± 0.67, and 3.50 ± 0.61, with an overall score of 3.40 ± 0.48. According to the physical therapists, all 32 patients were safely treated, and the main adverse effects reported by patients were fatigue and muscle pain, which was consistent with the findings of the patients' responses. Some patients complained of pain at the surgical site, which differed from the patients' responses. However, it was mild and did not exacerbate existing pain, or significantly interfere with ongoing treatment.

## 4 Discussion

This study has significance in proposing a new protocol utilizing RAGT for early rehabilitation therapy after spinal surgery. Also, it is the first to determine the effects and safety of early RAGT using this protocol after spinal surgery from both patient and physical therapist perspectives. Within ~POD 14, all patients underwent RAGT successfully, and none experienced any severe adverse events.

Wearable exoskeletal robots for RAGT have been implemented in various settings without standardized protocols (19). Hence, we modified and further developed a RAGT protocol. The draft protocol was primarily structured around a walking mode to enable patients to achieve independent gait function. However, using the initial draft protocol revealed several issues when training was primarily focused on gait mode. Firstly, adaptation to the robot was necessary from the perspective of the patients, as it was their first experience with RAGT. Therefore, we added a stand mode at the start of the protocol to facilitate adaptation. Furthermore, conditions such as sit-to-stand, weight shifting, and standing on one-leg needed to be fulfilled for gait to exist. Therefore, we added these modes before starting gait mode.

Table 1 shows that when patients undergo RAGT for the first time, they start with A mode, in which all stages require standing for 30 s as a basic requirement. If standing for 30 s is not difficult during stage 1 of this mode, the patient progresses to stage 2 of A mode and complete 20 squats (to enhance lower limb strength). If this is completed smoothly, the patient proceeds to stage 3 of A mode, which comprises >20 squats, but with a slower knee grading. Thus, A mode primarily focuses on muscle strengthening.

When A mode is completed, the patient progresses to B mode, which requires standing for 1 min as a basic requirement. In stage 1 of B mode, weight shifting is practiced by alternately lifting hands. Stage 2 of B mode is stair mode, which involves alternately lifting feet while shifting weight. Stage 3 of B mode is stair mode, which requires lifting one foot. Weight shifting is predominantly practiced in B mode, focusing on balance. After completing all stages of B mode, the patient progresses to C mode, all stages of which, train an even level gait. Additional assistive devices were applied for gait practice during the first and second stages of C mode. Steps and cadence settings were determined based on empirical observations, and aimed to achieve 200–400 steps with a cadence of < 50 if supported by a harness or walker, and 600–800 steps with a cadence > 70 for patients using crutches. After completing stage 1 of C mode, the second stage requires more steps. Upon successful completion of all previous modes and stages, the training proceeds to stage 3 of C mode, where either uneven level gait or up-and-down stairs is focused. This is practicing functional activities essential for daily life through RAGT. Thus, C mode training is primarily directed to enhancing endurance.

Patients underwent rehabilitation using the modified protocol described above, and the FAC and ambulation item of the MBI were measured. The results indicated that functional improvement was evident despite the limitations of a single-arm study and the short duration of RAGT rehabilitation. Therefore, we confirmed that the protocol used herein contributed to the functional recovery of patients. When creating the RAGT protocol, the most important considerations, in addition to the functional recovery of patients, were ensuring the protocol's convenience and effectiveness for both patients and the physical therapists administering RAGT. We then surveyed both groups to assess the training's effectiveness and convenience. Patient responses indicated satisfaction with the treatment, although feedback on both the robot and the effectiveness of RAGT was neutral. In contrast, the responses of the physical therapists who administered RAGT to the survey were positive, emphasizing the stability of RAGT and its effectiveness, except for its impact on their working environment.

Scores on the 5-point Likert scale ranging from 2.61–3.40 3.41–4.20, and >4.21 were classified as neutral, “agree,” and “strongly agree” (20). Accordingly, subsection items scoring >3.40 included “Noise,” four RAGT-related items (“easy to adapt to,” “improvement,” “future use,” and “recommendation”), as well as “fear of falling” and “walking ability” in terms of effectiveness. No items scored < 2.60, indicating “disagree,” across all aspects, including evaluation by the physical therapists. These survey results coupled with the absence of adverse events, affirmed the acceptability of the new introduced RAGT protocol for both patients and physical therapists, while also suggesting areas for improvement.

Neutral ratings for “Ease of wear” and “Comfort while wearing” within the “Related to robot” section aligned with previous findings. Challenges and discomfort associated with wearing the exoskeletal wearable robot were reported (17, 18). These issues might stem from unresolved postoperative pain when initiating RAGT soon after surgery, as well as the fact that the robot was not specifically designed for spinal surgery patients. Similarly, neutral ratings (2.65 and 2.63) for “Pain” and “Change positions” in the “Related to effectiveness” section might be attributed to postoperative pain concerns. However, under the supervision of physical therapists, and sometimes involving spinal orthosis, the 32 patients completed the treatment regimen without significant adverse symptoms. Although not explicitly evident in the survey data, discomfort and pain tended to become reduced as treatment progressed, indicating gradual improvement.

A notable finding was high satisfaction in terms of “Fear of falling” and “Walking ability.” This suggested that early rehabilitation soon after surgery through RAGT could expedite functional recovery by addressing concerns related to fear of falling. Given that patients undergoing spinal surgery often experience vague anxieties about gait (21), which can impede early mobility, RAGT can help to alleviate these fears by providing repetitive gait and targeted practices that were previously achievable only with the involvement of multiple therapists in conventional physical therapy (22). This is crucial to facilitate the functional recovery of patients and consequently reduce the burden of caregiving associated with rehabilitation.

Postoperative rehabilitation typically spans several months (23), suggesting that the five RAGT sessions in this study might have been insufficient. From the patient's perspective, it might have been challenging to anticipate or expect significant improvements in these areas after only five treatment sessions. This potentially resulted in relatively low ratings for “Muscle power” and “Balance”. Although the number of RAGT sessions was limited to five on average due to constraints in our institutional treatment setting, the primary aim of assessing RAGT feasibility in the early post-surgery phase was achieved, as evidenced by the improvement in functional evaluations at the time of transfer to the Department of Rehabilitation Medicine and at discharge. Accordingly, incorporating RAGT in addition to current conventional physical therapy appears beneficial for patient functional recovery. However, future studies should compare the effects of RAGT over a longer term and also compare its effects with conventional physical therapy.

Alleviating the workload of treatment providers is crucial when introducing and expanding new treatments, especially for physical therapists who have a high mental workload (24). In this context, the physical therapists received a comprehensive education about RAGT before its implication. We piloted RAGT with five patients before the survey study. However, from the perspective of physical therapists providing RAGT, they reported low scores in the “Work Environment” category. This could be attributed to the novelty of the treatment and the new program, as the physical therapists were unfamiliar with its intricacies. It is anticipated that this situation will improve with continued RAGT application and ongoing education initiatives.

This study has several limitations. First, the small sample size, further subdivided by the surgical levels and types, resulted in even smaller groups. This could have led to an overestimation of satisfaction levels, as patients with higher satisfaction may have been more likely to participate in the survey. However, the primary goal of this study was to introduce a new RAGT protocol for post-spinal surgery rehabilitation and assess its feasibility through patient satisfaction. The confirmation of this protocol's feasibility is a meaningful outcome. As RAGT becomes more widely used, the number of participating patients should increase, allowing for more detailed satisfaction surveys and protocol refinements. Second, the study included only five therapists, suggesting a need for further research with a larger therapist cohort. Third, the short treatment period is another limitation, influenced by the constraints of our hospital. Longer-term studies in other hospitals could provide more comprehensive evaluations of the protocol's effectiveness and patient satisfaction over time. Fourth, the study did not fully investigate whether the RAGT protocol used is the optimal treatment approach. Although the protocol has undergone three revisions, further research is necessary to thoroughly assess its effectiveness and appropriateness. Adjustments to the robot and improvements to the protocol are needed to better meet the needs of patients after spinal surgery. Lastly, the study did not include a comparison with a group that received only conventional therapy. While patients in this study showed functional improvement, comparing the outcomes between those who received only conventional therapy and those who received RAGT in addition would have provided a clearer understanding of RAGT's effectiveness. These factors limit the generalizability of the study's results. Despite these challenges, the study provides significant value in establishing a foundation and direction for future research.

In conclusion, this study introduced a new RAGT protocol for early rehabilitation after spinal surgery and confirmed its effectiveness in providing repetitive gait training without significant side effects. The protocol also showed high patient satisfaction, reducing fears of falling and potentially leading to better functional outcomes. Physical therapists found RAGT to be a safe and satisfactory method. These findings suggest that RAGT could serve as an additional treatment option alongside conventional physical therapy. However, further development is needed as more patients and therapists gain experience with the protocol, and it should be refined through discussions among healthcare professionals. Practical considerations, such as improving therapists' working conditions, should also be addressed.

## Data Availability

The raw data supporting the conclusions of this article will be made available by the authors, without undue reservation.
