# Supplementary material for: New protocol for early robot-assisted gait training after spinal surgery
Source: Front Med (Lausanne). 2024 Oct 23;11:1450883. doi: 10.3389/fmed.2024.1450883 (PMC11539955; doi:10.3389/fmed.2024.1450883)
Supplement: Supplementary file 2 [file Data_Sheet_2.docx]

**Supplementary table 1. First version of protocol for early robot-assisted gait training after spinal surgery.**

| **Mode** | **Stage** | | |
| --- | --- | --- | --- |
|  | **1** | **2** | **3** |
| **A** | Stand for 30 seconds (stand mode) | Stand for 30 seconds (stand mode) | Stand for 30 seconds (stand mode) |
|  |  | + Squat training < 100 times (squat mode) | + Squat training > 100 times (squat mode) |
| **B** | Even level gait training  (gait mode)  Steps: < 500,  Cadence: < 50 | Even level gait training  (gait mode)  Steps: 500‒ 700,  Cadence: < 50 | Even level gait training  (gait mode)  Steps: 700‒1,000,  Cadence: < 65 |
| **C** | Even level gait training  (gait mode) | Even level gait training  (gait mode) | Even level gait training  (gait mode) |
|  | (Independent)  Steps: 500‒800  Cadence: < 50  [Crutch]  Steps: 200‒400  Cadence: > 70 | (Independent)  Steps: 700‒1,000  Cadence: < 65  [Crutch]  Steps: > 1,500  Cadence: > 75 | (Independent)  Steps: > 1,000  Cadence: > 70  [Crutch]  Steps: > 1,500  Cadence: > 75 |
|  |  |  | + Uneven level attempt  (gait mode and  up-and-down stairs mode) |

The robot is worn for 5 minutes. Each session, involves A + B or C modes, totaling 25 minutes. Selected modes and stages might vary depending on condition of each patient. Assistive devices comprising harness with anterior walker, anterior walker alone, or crutches can be used in mode B based on functional status of patients. Depending on their ability, patients can walk independently with robot assistance or use crutches alongside robot in mode C.

**Supplementary table 2. Second version of protocol for early robot-assisted gait training following spinal surgery.**

| **Mode** | **Stage** | | |
| --- | --- | --- | --- |
|  | **1** | **2** | **3** |
| **A** | Stand for 30 seconds (stand mode) | Stand for 30 seconds (stand mode) | Stand for 30 seconds (stand mode) |
|  |  | + 20 squats  (squat mode) | + > 20 squats  (squat mode) |
| **B** | Even level gait training  (gait mode)  Steps: < 200,  Cadence: < 50 | Even level gait training  (gait mode)  Steps: 200 ~ 400,  Cadence: < 50 | Even level gait training  (gait mode)  Steps: > 400,  Cadence: < 65 |
| **C** | Even level gait  (gait mode) | Even level gait  (gait mode) | Even level gait  (gait mode) |
|  | (Independent)  Steps: 200‒400  Cadence: < 50  [Crutch]  Steps: 600 ~ 800  Cadence: > 70 | (Independent)  Steps: 400‒600  Cadence: < 65  [Crutch]  Steps: > 800  Cadence: > 75 | (Independent)  Steps: > 600  Cadence: > 70  [Crutch]  Steps: > 800  Cadence: > 80 |
|  |  |  | + Uneven level try  (gait mode and  Stair up and -down mode) |

The robot is worn for 5 minutes. Each session, involves A + B or C modes, totaling 25 minutes. Selected modes and stages might vary depending on condition of each patient. Assistive devices comprising harness with anterior walker, anterior walker alone, or crutches can be used in mode B based on functional status of patients. Depending on their ability, patients can walk independently with robot assistance or use crutches alongside robot in mode C.

The changes from the first version: In B mode 1 stage, we reduced the step count from under 500 to under 200 after realizing that patients couldn’t complete the planned number of steps. Similar adjustments were made in C mode 1 stage. For patients using crutches, who generally have a higher functional level, we increased the step count from 200-400 to 600-800. In A mode 2 stage, we reduced squat repetitions from under 100 to under 20 based on patient performance. Step counts in B and C mode 2 stages were also adjusted according to patients' abilities. In A mode 3 stage, we reduced squat repetitions from over 100 to over 20, with similar adjustments in B and C mode 3 stages.

**Supplementary table 3. Third version of protocol for early Robot-Assisted Gait Training following spinal surgery.**

| **Mode** | **Stage** | | |
| --- | --- | --- | --- |
|  | **1** | **2** | **3** |
| **A** | Stand for 30 seconds (stand mode) | Stand for 30 seconds (stand mode) | Stand for 30 seconds (stand mode) |
|  |  | + 20 squats (squat mode) | + > 20 squats with slow sitting down (squat mode) |
| **B** | Stand training for 1 minute (stand mode) | Stand for 1 minute (stand mode) | Stand for 1 minute (stand mode) |
|  | + Weight shifts  lifting hands (stand mode) | + Weight shifts  lifting feet alternately (stair mode) | + Weight shift training lifting one foot (stair mode) |
| **C** | Even level gait training  (gait mode) | Even level gait training  (gait mode) | Even level gait training  (gait mode) |
|  | (Harness or Walker)  Steps: 200‒400  Cadence: < 50  [Crutch]  Steps: 600‒800  Cadence: > 70 | (Harness or Walker)  Steps: 400‒600  Cadence: < 65  [Crutch]  Steps: > 800  Cadence: > 75 | (Harness or Walker)  Steps: > 600  Cadence: > 70  [Crutch]  Steps: > 800  Cadence: > 80 |
|  |  |  | + Uneven level try  (gait mode and  stair-up and -down mode) |

The robot is worn 5 minutes. Each session, involves A + B or C modes, totaling 25 minutes. Selected modes and stages might vary depending on condition of patient. Assistive devices comprising harness with anterior walker, anterior walker alone, or crutches can be used in mode B based on functional status of patients. Depending on their ability, patients can walk independently with robot assistance or use crutches alongside robot in mode C.

The changes from the second version: In A mode 3 stage, we clarified "slow sitting down" to "squat" for accuracy. In B mode, we shifted from counting steps to measuring training time, focusing on the maximum time patients could perform. In C mode, we replaced independent execution with the use of a harness or walker to ensure safety, even for those capable of performing independently.
